# The Antseptic Ligature, as Applied in the Operation for Varicocele

**Published:** 1871-08

**Authors:** Emile Steiger

**Affiliations:** Prairie du Chien, Wis.


### THE ANTISEPTIC LIGATURE, AS APPLIED IN THE OPERATION FOR VARICOCELE.

BY EMILE STEIGER, M.D., Prairie du Chien, Wis.

The operation for varicocele and circocele was heretofore considered most dangerous, and was performed principally in three different methods: 1. Dissection of the spermatic bundle and ligature of the varicose veins. 2. Compression by Breschet's pincers. 3. Subcutaneous ligature, or the proceedings of "Gagnebe."

Particularly the first method, but more or less also the second and third, were often followed by dangerous, even fatal phlebitis. Involuntary compression or ligation of the spermatic artery, and consecutive atrophy of the testicle, endangered the second and third method.

I was called in consultation by my friend, Dr. Martin, of McGregor, in the case of L. S. (case of circocele), and I believed that ligation of spermatic artery and atrophy of the testicle could be avoided by free dissection, while phlebitis could be prevented through the antiseptic effect of carbolic acid.

The operation was performed on the 3d of April, 1871, in the following manner:

1. Incision on the left side of the scrotum, about one inch long, so as to lay open the bundle formed by the fascia of the cremaster.

2. Fixing of the bundle outside of the external wound, with help of a sonde passed under it, across and over the margins of the scrotal incision.

3. Dissection of the cord, and application of two antiseptic ligatures around the varicose vein, separated nearly one inch, and cut close to the knot.

4. Excision of that portion of the vein included between the two ligatures.

5. Careful cleansing of all parts with a solution of carbolic acid j., glycerine iv, aq. xij.., by sponge and syringe.

6. Closing of external wound with silk sutures, which had been prepared by being soaked for six hours in carbolated oil, and covering the scrotum with linen, also soaked in carbolated oil.

Two weeks after the operation patient was well, happy, and attending to business.

I owe to the kind suggestion of Dr. D. Mason my preparation of antiseptic ligatures, consisting of violin strings, kept in a bottle of carbolated oil for at least 48 hours.

